# *Tor1a*+/- mice develop dystonia-like movements via a striatal dopaminergic dysregulation triggered by peripheral nerve injury

**DOI:** 10.1186/s40478-016-0375-7

**Published:** 2016-10-03

**Authors:** Chi Wang Ip, Ioannis U. Isaias, Burak B. Tekin, Dennis Klein, Janos Groh, Aet O’Leary, Susanne Knorr, Takahiro Higuchi, James B. Koprich, Jonathan M. Brotchie, Klaus V. Toyka, Andreas Reif, Jens Volkmann

**Affiliations:** 1 Department of Neurology, University Hospital of Würzburg, University of Würzburg, Josef-Schneider-Strasse 11, 97080 Würzburg, Germany; 2 Department of Psychiatry, Psychosomatics and Psychotherapy, Goethe-University Frankfurt, Frankfurt am Main, Germany; 3 Department of Nuclear Medicine, University Hospital of Würzburg, University of Würzburg, Oberdürrbacher Strasse 6, 97080 Würzburg, Germany; 4 Toronto Western Hospital, University Health Network, 100 King St W, Suite 5600, Toronto, Canada

**Keywords:** Dystonia, DYT1, Dopamine, Peripheral injury, Second hit

## Abstract

Isolated generalized dystonia is a central motor network disorder characterized by twisted movements or postures. The most frequent genetic cause is a GAG deletion in the *Tor1a* (DYT1) gene encoding torsinA with a reduced penetrance of 30-40 % suggesting additional genetic or environmental modifiers. Development of dystonia-like movements after a standardized peripheral nerve crush lesion in wild type (wt) and *Tor1a+/-* mice, that express 50 % torsinA only, was assessed by scoring of hindlimb movements during tail suspension, by rotarod testing and by computer-assisted gait analysis. Western blot analysis was performed for dopamine transporter (DAT), D1 and D2 receptors from striatal and quantitative RT-PCR analysis for DAT from midbrain dissections. Autoradiography was used to assess the functional DAT binding in striatum. Striatal dopamine and its metabolites were analyzed by high performance liquid chromatography. After nerve crush injury, we found abnormal posturing in the lesioned hindlimb of both mutant and wt mice indicating the profound influence of the nerve lesion (15x vs. 12x relative to control) resembling human peripheral pseudodystonia. In mutant mice the phenotypic abnormalities were increased by about 40 % (*p* < 0.05). This was accompanied by complex alterations of striatal dopamine homeostasis. Pharmacological blockade of dopamine synthesis reduced severity of dystonia-like movements, whereas treatment with L-Dopa aggravated these but only in mutant mice suggesting a DYT1 related central component relevant to the development of abnormal involuntary movements. Our findings suggest that upon peripheral nerve injury reduced torsinA concentration and environmental stressors may act in concert in causing the central motor network dysfunction of DYT1 dystonia.

**Electronic supplementary material:**

The online version of this article (doi:10.1186/s40478-016-0375-7) contains supplementary material, which is available to authorized users.

## Introduction

Dystonia is a clinical syndrome characterized by sustained or intermittent muscle contractions causing abnormal, often repetitive, movements, postures, or both. According to the most recent consensus-based definition, dystonic movements are typically patterned and twisting, may be tremulous and are often initiated or worsened by voluntary action and associated with overflow muscle activation [2]. Epidemiological studies implicate that more than 3 million people worldwide are suffering from dystonia [25].

Etiologically, dystonia has multiple acquired and genetic factors most likely converging to a multistep pathophysiological pathway leading to a central motor network disorder with a dystonic phenotype [26, 37]. DYT1 is the most common form of inherited dystonia and is linked to a GAG deletion on the torsinA encoding gene *Tor1a*. The penetrance of the typical human limb onset generalized DYT1 dystonia phenotype is present in only 30-40 % of gene carriers [28]. Because dystonia can also be caused by a trauma [24, 51, 57] or by repetitive limb overuse, and in the absence of a known genetic cause, the “two-hit” hypothesis for developing dystonia has emerged. This is thought to involve intrinsic predisposition and environmental triggers acting in concert [30]. In a pharmacological mouse model of DYT12 dystonia the dystonic phenotype was provoked by electrical stress [8]. A non-genetic animal model for blepharospasm was created by combined toxin induced striatal dopamine (DA) depletion and lesioning of the zygomatic nerve [46].

We hypothesized that a peripheral nerve injury temporarily disrupting sensorimotor integration in the affected limb may elicit focal dystonia in genetically predisposed individuals. Several genetic alterations of the *Tor1a* gene have been introduced into mice generating models for DYT1 dystonia. These animals either lack the typical dystonic phenotype [49, 53] or have only subtle motor deficits dissimilar to the human clinical presentation [14, 20] or they suffer from neurodegeneration [31, 38] unlike human DYT1 brains at post-mortem [17, 39, 45]. To investigate the role of peripheral stressors we specifically chose the *Tor1a+/-* mouse model since it does not develop overt dystonia nor any signs of neurodegeneration. It has been shown that the *Tor1a+/-* mouse produces only 50 % torsinA and that the mutated torsinA exhibits loss of function [19, 54]. We here demonstrate that a peripheral nerve lesion can elicit dystonia-like movements in wild type (wt) and mutant mice. Only *Tor1a+/-* mice, however, demonstrate a centrally mediated component of dystonia-like movements as indicated by several biomarkers of striatal dopaminergic dysregulation and by showing a treatment response to DA depletion therapy.

## Materials and methods

### Animals

Heterozygous *Tor1a* knockout mice (*Tor1a*+/-) [19] and wild type (wt) littermates of a mixed C57BL/6 J and 129 background were purchased from the Jackson Laboratory (described as B6; 129-Tor1atm1Wtd/J, strain 006251) and bred in our animal facility. Determination of the genotype was performed by PCR according to the protocol from the Jackson Laboratory using the following primers: a) mutant forward: CGGTCGCTACCATTACCAGT; b) wt forward: GCTGGCACGCCTTATTACTC. A common reverse primer was used: TAGAGCTCTGGGCTTGGAAA. These primers resulted in following band sizes: knockout 1600 bp, wt 308 bp (Additional file 1: Fig. S1 a). Each PCR was performed in a volume of 25 μl using a Hot Start PCR Master Mix. We used the following PCR protocol: 95 °C, 5 min; 95 °C, 30 s; 65 °C, 1 min; 72 °C, 2 min; 39 cycles, 72 °C, 5 min. Analyses were performed on mice with an average body weight of 25 g investigated at the age of 4 months. Mice were not randomized and were selected on availability including both male and female (Additional file 1: Fig. S1 b). Possible gender differences were analyzed post hoc, but no significant difference was found in any of the experiments.

### Behavioral studies

#### Severity rating of dystonia-like movements

Frequency and duration of dystonia-like movements of the affected right hindlimb were assessed during a 30 s tail suspension test using a self-developed 0-4 point scoring system (dystonia-like movement scale; DLMS). The DLMS was rated from video clips (30 s) of the tail suspension test by two observers (C.W.I. and B.B.T.) blinded to the group assignment and timepoint to assure an objective outcome assessment. Reported DLMS scores reflect the mean of the two independent ratings: 0: no abnormal movement (Additional file 2: Suppl. video 1); 1: short hindlimb retraction and clenching of the foot only once during 30 s period (Additional file 3: Suppl. video 2); 2: repeated hindlimb retraction and clenching of the foot each episode lasting < 1 s (Additional file 4: Suppl. video 3); 3: repeated hindlimb retraction and foot clenching episodes lasting ≥ 1 and < 2 s. 4 (Additional file 5: Suppl. video 4): repeated hindlimb retraction and foot clenching with episodes lasting ≥ 2 s and hindlimb drawn up to the abdomen (Additional file 6: Suppl. video 5).

#### Rotarod performance test

Mice were tested 3 times per session on a rotarod (RotaRod Advanced, TSE systems) with accelerating speed from 5 to 50 rpm for up to 300 s. Animal falls and latencies to fall were recorded.

#### CatWalk XT gait analysis

Mice were placed on one side of the CatWalk XT (Noldus, Wageningen, Netherlands) transparent glass runway and were motivated by food pellet rewards to run to the other side. Gait and footprints were recorded by a video camera located underneath the animals. Three runs per animal were analyzed (program version 10.0.408). Animals were excluded from gait analysis if they stopped when walking through the area of recording.

### Sciatic nerve crush injury

Under deep anesthesia with ketamine-xylazine the gluteal region of the right hind paw was shaved and incised under sterile condition. The right sciatic nerve was exposed and crushed at the region of the sciatic notch by using a non-serrated clamp that was placed around the isolated nerve with a constant and reproducible pressure for 30 s. Sham operated animals received the same surgery but without crush.

### Injection of L-3,4-dihydroxyphenylalanine (L-Dopa)/benserazide and alpha-methyl-p-tyrosine (AMPT)

AMPT dissolved in normal saline was i.p. injected three times within 24 h once weekly until week 8 at a dose of 100 mg/kg bodyweight per injection 24, 20 and 4 h before behavioral testing was done as described [55]. Benserazide, a DOPA decarboxylase inhibitor, was dissolved in saline and i.p. injected at a dose of 12 mg/kg/day 15 min before i.p. administration of 30 mg/kg/day L-Dopa once daily as described [52] until week 8 after sciatic crush.

### Nerve conduction studies

Recordings were done on a digital Neurosoft-Evidence 3102 electromyograph (Schreiber & Tholen Medizintechnik) as described [29]. In brief, after i.p. anaesthesia with ketamine/xylazine (10:1; 10 μl/g body weight) supramaximal stimulation of the tibial nerve was done with needle electrodes above the ankle (distal) and of the sciatic nerve at the sciatic notch proximal to the lesion site (about 34 °C skin temperature). Compound muscle action potentials (CMAP) were recorded at the foot muscles with steel needle electrodes. Peak to peak CMAP amplitudes were determined. Latencies were measured and the corresponding nerve conduction velocities (NCV) were calculated. The investigators (D.K. and K.V.T.) were not aware of the genotype of the analyzed mice.

### Immunohistochemistry and tissue analysis

Eight weeks after nerve crush or sham operation mice were transcardially perfused with 0.1 M phosphate buffered saline (PBS) at room temperature (RT). Freshly dissected mouse brain, lumbar spinal cord (L2-L4) and right sciatic nerve were snap frozen in liquid nitrogen-cooled isopentane. Ten μm transverse cryosections of the sciatic nerve, the spinal cord and coronal sections of the brain at the region of 0.38 mm relative to the bregma were cut for further staining (Paxinos and Franklin, The Mouse Brain in Stereotaxic Coordinates, 2001, Fig. 28). The following antibodies were applied before using the ABC-system (Dako, Hamburg, Germany) with 3,30-diaminobenzidine as peroxidase substrate: chicken anti mouse MPZ (myelin protein zero) (Acris Antibodies, Rockville, USA), mouse anti neurofilament 68 kDa (Sigma-Aldrich, Munich, Germany), rat anti mouse F4/80 and rat anti mouse CD11b (Serotec, Oxford, UK). Specificity of the immunoreaction was assessed by omission of the primary antibody.

For Nissl stains spinal cord, cerebral cortex and striatal sections were immersion fixed in 0.1 M PBS containing 4 % paraformaldehyde for 10 min followed by incubation with 0.1 % cresyl violet for 10 min, rinsing, dehydration and mounting for light microscopy.

Quantification of the number of macrophages, microglia and neurons was performed with a light microscope (Olympus BH2, Olympus, Hamburg, Germany) using an ocular grid covering a defined area (0.0256 mm^2^) at a final magnification of 600×. Neurofilament and MPZ staining intensities were measured by optical densitometry using the MetaVue program, version 6.3r2 (Visitron Systems, Munich, Germany).

### Western blot analyses

After transcardial perfusion with PBS both striata were dissected and snap frozen in liquid nitrogen. Tissue was sonicated in radioimmunoprecipitation assay lysis buffer (25 mM Tris-HCl pH 8, 10 mM Hepes, 150 mM NaCl, 145 mM KCl, 5 mM MgCl2, 2 mM EDTA, 0.1 % sodium dodecyl sulphate, 1 % NP-40, 10 % glycerol). Protein concentration was determined by Lowry assay and proteins were resolved by sodium dodecyl sulphate-polyacrylamide gel electrophoresis, transferred to nitrocellulose membranes and visualized using Ponceau S. Membranes were blocked with Roti®-Block (Carl Roth) and probed with respective antibody solutions overnight at 4 °C (DAT, Millipore MAB369; D1 receptor, Santa Cruz, sc-1434; D2 receptor, Millipore, AB5084P; GAPDH, abcam, ab9484). Incubation with horseradish peroxidase-conjugated secondary antibodies was performed for 1 h at RT and detection was achieved by use of ECL reagent and ECL hyperfilm (GE Healthcare Bio-Sciences AB). Sequential stainings were performed after incubating the nitrocellulose membrane with stripping buffer (0.2 M glycine, 0.1 % sodium dodecyl sulphate, 10 mM dithiothreitol, and 1 % Tween) for 30–120 min. Completeness of removal of the first set of primary antibodies was controlled by staining with secondary antibodies. Different exposure times of ECL hyperfilms were tested and the resulting signals were quantified in the linear range by densitometry using NIH ImageJ software. Results were normalized to naïve wt littermate protein level as a reference and related to GAPDH as loading control.

### Semi-quantitative real-time PCR

Snap frozen midbrain was homogenized (ART-MICCRA D-8, ART Labortechnik) in TRIzol® reagent. Total RNA was isolated according to the guidelines of manufacturers. Concentration and quality of RNA was determined using a BioPhotometer and 1 μg of RNA was reverse transcribed in a 100 μl reaction using random hexamer primers. Complementary DNA samples were subsequently analyzed as triplicates by semi-quanitiative real-time polymerase chain reaction using pre-developed TaqMan® assays (Mm00438388_m1) and TaqMan® universal PCR master mix (Applied Biosystems). Results were normalized to a reference naïve wt mRNA level.

### Autoradiography

FP-CIT (0.74 MBq) was injected via tail vein 20 min before euthanasia. The brain was taken out, then immediately frozen and cut into 20-μm coronal slices. Autoradiography plates were exposed to the slices immediately for 45 min for visualization of FP-CIT distribution with a digital autoradiography system (CR 35 Bio; Raytest). In order to quantify tracer uptake distribution, regions of interests (ROIs) were drawn manually at the striatum.

### Striatal dopamine analysis by high performance liquid chromatography (HPLC)

Striata were homogenized with Branson Digital Sonifier (G. Heinemann Ultraschall- und Labortechnik, Schwäbisch-Gmünd, Germany) in ice-cold aqueous solution of H3PO4 (150 mM) and DTPA (500 μM). The homogenate was then centrifuged at 40700 g for 20 min at 4 °C. Aliquots (50 μl) of the obtained supernatant were chromatographed on a Nucleosil 100-5 C18 column (250 mm x 4.6 mm; 5 μm) (Macherey-Nagel, Düren, Germany). The separation was done in isocratic elution mode at room temperature using mobile phase containing 0.02 M sodium citrate, 0.1 mM EDTA, 0.01 M sodium phosphate, 0.003 M octanesulphonic acid, 0.003 M heptanesulphonic acid, 7 % acetonitrile, and 3 % methanol at a pH adjusted to 3.1 with diethylamine. For external standard, a stock solution containing 500 μg/ml dopamine, homovanillic acid, and 3,4-dihydrophenylacetic acid (Sigma-Aldrich, Steinheim, Germany) was prepared. The chromatography system consisted of an Agilent 1100 Series isocratic pump, a thermostatted autosampler, a thermostatted column compartment and a Bio-Rad 1640 electrochemical detector with glassy carbon electrode. The measurements were done at an electrode potential of +0.72 V versus the Ag/AgCl reference electrode. Results were normalized to reference naïve wt neurotransmitter level.

### Statistical analysis

**Fig. 1:**
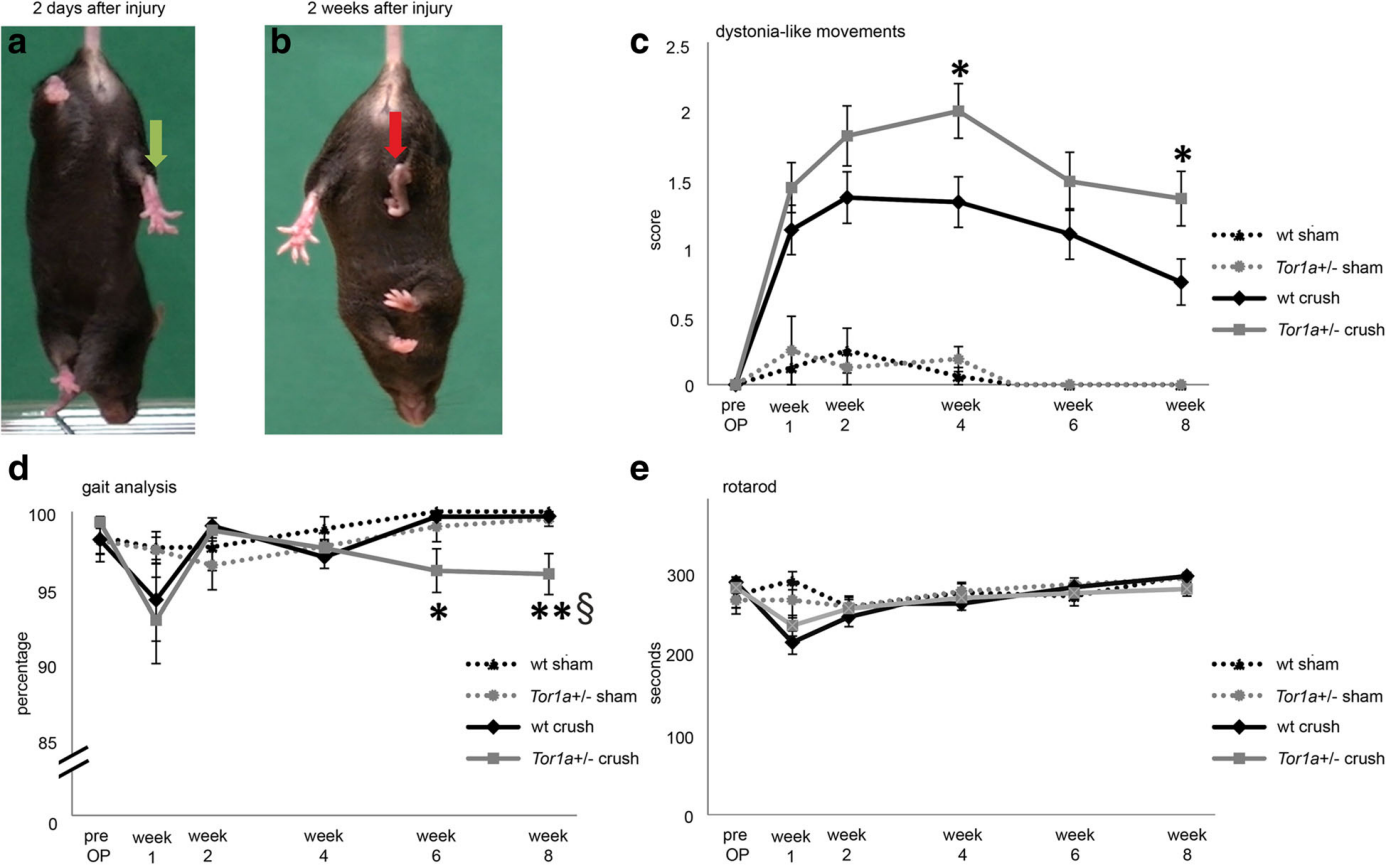
Tail suspension test shows focal dystonia-like movements in wt and *Tor1+/-* mice that is induced by sciatic nerve crush. **a, b** Images of a *Tor1a*+/- mouse 2 days and 2 weeks after sciatic nerve crush of the right hind leg. Typical defect is shown when muscles of the affected leg are still profoundly weak. Therefore, the degree of dystonia-like postures (**b**) may be even underrated at this early timepoint of 2 weeks. **c** Temporal evolution of abnormal movements on the DMLS of the right hind leg measured during tail suspension in crush-injured *Tor1a*+/- mice (grey line; *n* = 33) and wt mice (black line; *n* = 30) as well as sham operated *Tor1a*+/- (dotted grey line; *n* = 8) and wt mice (dotted black line; *n* = 8) during an observation time of 8 weeks. Mean values for each time point ± SEM are shown. **d** Gait analysis displaying the step sequence regularity index (percentage) as measurement of the interpaw-coordination during ambulation in crush-injured *Tor1a*+/- (grey line; *n* = 30) and wt mice (black line; *n* = 23) as well as sham operated *Tor1a*+/- (dotted grey line; *n* = 8) and wt mice (dotted black line *n* = 8)(mean ± SEM). Before sciatic nerve injury a normal step sequence regularity index in both genotypes was observed, reflecting a normal interpaw-coordination during ambulation. One week after trauma the regularity index decreased in all groups with greater impairment in crush injured than in sham operated mice. While sham operated wt or *Tor1a*+/- mice as well as crush injured wt mice recovered with time, the *Tor1a*+/- crush group did not, leading to a significant reduction of step sequence regularity index at week 6 an 8 (*p* < 0.05; *p* < 0.01 respectively) compared to crush injured wt mice. **e** Diagram showing results of the rotarod performance test (grey line, *Tor1a*+/- crush *n* = 23; black line, wt crush *n* = 19; dotted grey line, *Tor1a*+/- sham *n* = 8, dotted black line, wt sham *n* = 8)(mean ± SEM). The motor impairment of *Tor1a*+/- mice after recovering from the nerve crush was subtle during spontaneous motor behavior and did not manifest in a rotarod analysis. * and ** denote significant differences (*p* < 0.05 and *p* < 0.01 respectively) comparing nerve injured *Tor1a+/-* and wt mice for one time point (non-parametric two-tailed Mann-Whitney test). § demonstrates a significant difference after Bonferroni-Holm correction of the p-values for the whole time span of 8 weeks

For statistical analysis of behavioral data, the distribution of the values was investigated via Q-Q-plots. None of the plots showed normal distribution, thus non-parametric methods were employed as statistical tests. To compare two groups for each timepoint, the Mann-Whitney Test was used. As several time points were investigated Bonferroni-Holm correction was applied (§). To implement the change over time into the statistical analyses, we calculated the difference to the pre-operative values. For the figures, mean values ± SEM intervals as error bars are shown. Additionally, Cohens d as effect size measure was calculated for interpretation of the size of the effect in Fig. 1c. To interpret Cohens d data, values lower than 0.5 show a small effect, values between 0.5 to 0.8 represent a medium effect and values higher than 0.8 imply a large effect [12]. Statistical analysis was done with software R version 3.2.2. *p < 0.05, **p < 0.01, ***p < 0.001 were considered as significant *p*-values.

For data examination of immunohistochemistry, protein analysis and rt-PCR analysis we used the parametric one-way ANOVA with posthoc Tukey test. *p < 0.05, **p < 0.01, ***p < 0.001 were considered as significant *p*-values.

## Results

### *Tor1a*+/- mice develop more severe dystonia-like movements than wt mice after sciatic nerve crush

Two days after nerve injury, tail suspension tests revealed severe weakness of sciatic nerve innervated muscles leading to extension of the right hind leg (Fig. 1a). At later time points, repetitive, involuntary muscle contractions with clenching of the toes and retraction of the affected leg were detected in both wt and *Tor1a*+/- mice resembling focal dystonia-like movements (Fig. 1b) with a peak at four weeks after surgery followed by a continuous slow decrease of the DLMS in both genotypes. The score values were significantly higher as compared to sham operated controls that did not reveal abnormal movements and posturing (Fig. 1c). However, the frequency and duration of dystonia-like movements after nerve injury was higher in *Tor1a*+/- mutants as compared to wt mice with statistical significance at weeks four and eight after surgery (*p* < 0.05). Calculation of the effect size by Cohens d showed a small to medium effect of the genetic mutation in *Tor1a*+/- mice.

Despite the initial profound weakness due to the peripheral nerve lesion gait analyses demonstrated a slight but significantly impaired interpaw coordination in mutant mice as compared to wt mice at week 6 and 8 using the CatWalk system, while the overall motor performance on the rotarod test was rather mildly impaired in both genotypes showing no differences between both groups. Because of the complexity of the rodent walking pattern, even subtle changes in the motor performance of mice with dystonia-like movements could be detected demonstrating that kinematic gait analysis is the most appropriate method for quantifying the motor phenotype (Fig. 1d,e).

### Structural and functional recovery of the sciatic nerve is not different after crush injury between *Tor1a+/-* and wt mice

**Fig. 2:**
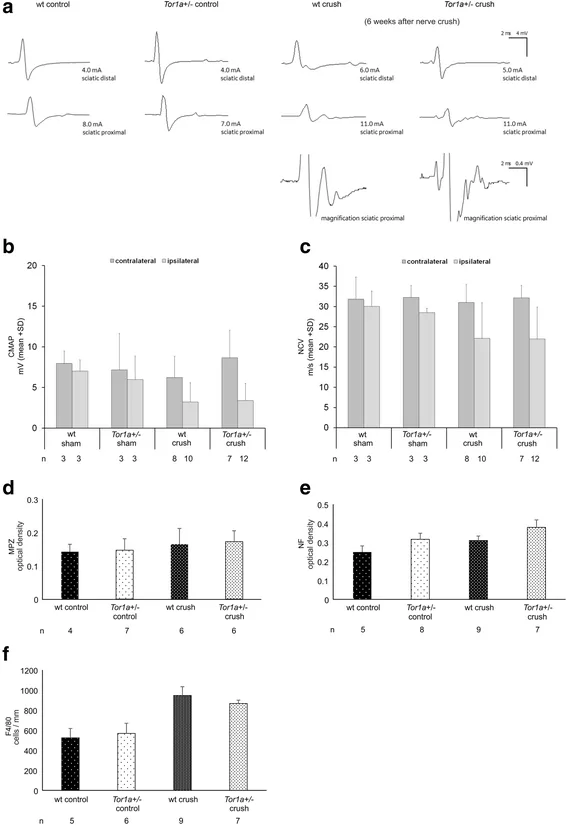
*Tor1a+/-* and wt mice do not show structural differences in the peripheral nervous system, spinal cord and brain. **a** Representative examples of a nerve conduction study in wt and *Tor1a*+/- control (left) and crush injured (right) mouse sciatic nerves. Magnification of proximal compound action potential in wt and *Tor1a*+/- crush injured mice illustrates abundant polyphasic late potentials indicating slowly conducting nerve fibre populations. **b, c** Functional analysis of sciatic nerves by nerve conduction study at the operated ipsilateral (light grey) and the unoperated contralateral (dark grey) side in *Tor1a*+/- and wt mice with sciatic nerve crush or sham injury. Parameters of interest (mean ± SD) are (**b**) compound muscle action potential (CMAP) and (**c**) the nerve conduction velocity (NCV). **d, e** Diagrams showing optical density of immunohistochemical staining against (**d**) myelin protein zero (MPZ) and (**e**) neurofilament (NF) (mean ± SEM). **f** Diagram showing number of F4/80+ macrophages/mm^2^ (mean ± SEM) in sciatic nerves of naïve wt, naïve *Tor1a*+/-, crush injured wt and *Tor1a*+/- mice. *n* = number of mice are depicted below the diagrams. Statistical analysis was performed by using the parametric one-way ANOVA with posthoc Tukey test

Baseline electrophysiological and immunohistological analysis comparing naïve wt with *Tor1a*+/- mice did not reveal any differences in sciatic nerve structure or function. Three days after nerve crush a complete conduction failure across the lesion was found in sciatic nerves of either genotype (data not shown). After six weeks compound action potential amplitudes (CMAP) as well as nerve conduction velocities (NCV) had recovered in wt and mutant mice by about half the pre-crush level with numerous low amplitude late and polyphasic potentials still present (Fig. 2a-c). Although CMAPs and NCVs were still lower at this time point as compared to control mice, the differences were not significant. There was no significant reduction of CMAP or NCV in sham operated mice (Fig. 2b,c). In addition immunohistochemical stainings of naïve wt and *Tor1a*+/- mouse sciatic nerves displayed the same optical density of myelin protein zero (MPZ)(Fig. 2d) and neurofilament (NF)(Fig. 2e) without obvious changes eight weeks after crush injury. Analyses of inflammatory cell numbers in wt and *Tor1a*+/- mice sciatic nerves displayed a similar trend but non-significant increase of F4/80+ macrophages in wt and mutant mice eight weeks after crush injury as compared to naïve animals (Fig. 2f).

### Neuronal cell density is unchanged in *Tor1a*+/- as compared to wt mouse spinal cord and brain

**Fig. 3:**
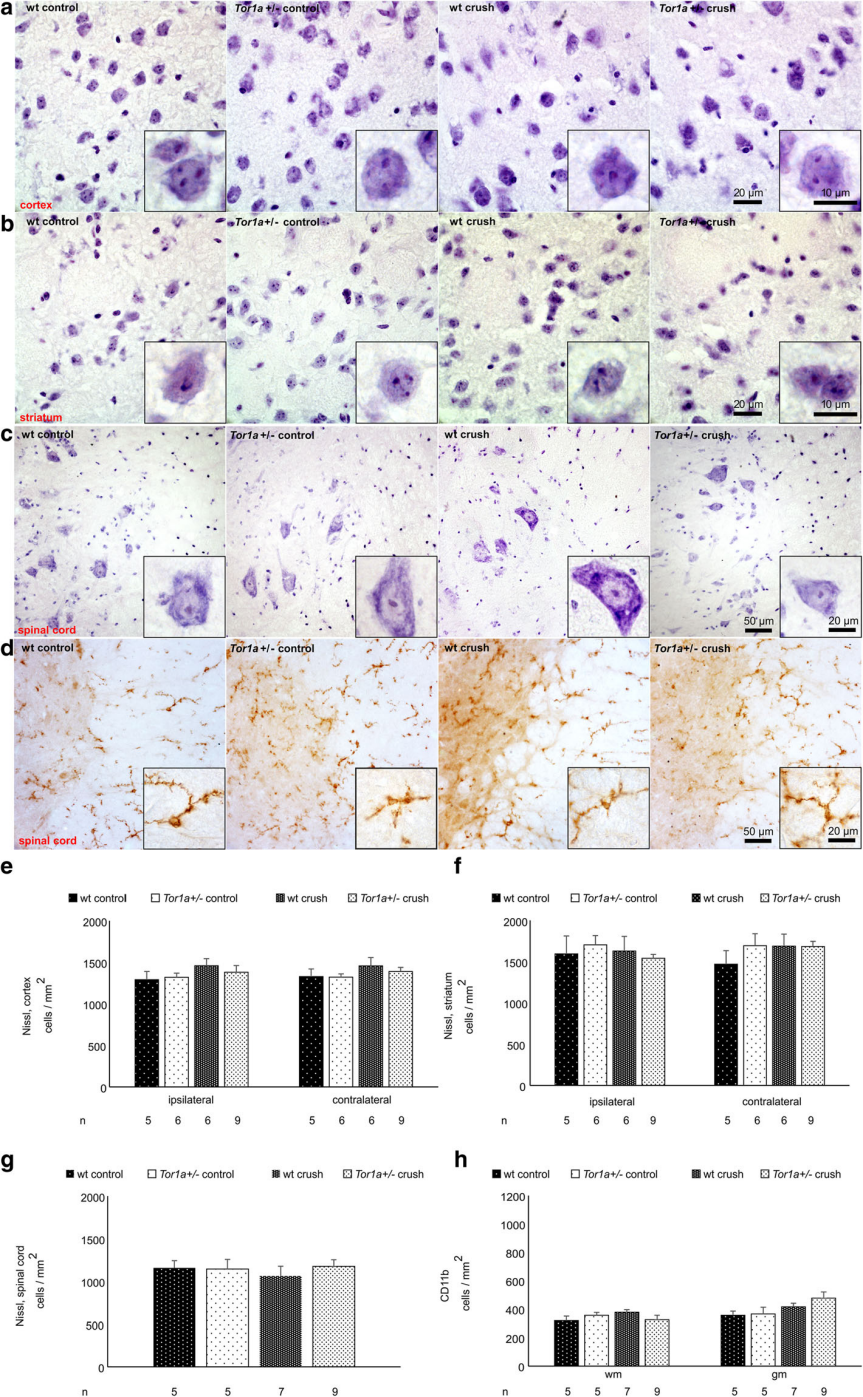
Tor1a+/- mice do not present morphological changes in brain and spinal cord after crush injury. **a-c** Representative images of Nissl stained neurons in (**a**) somatosensory cortex, (**b**) striatum and (**c**) spinal cord of naïve wt, naive *Tor1a*+/-, wt crush and *Tor1a*+/- crush injured mice. High magnification inserts of single cells are included in each image. **d** Representative images of CD11b + microglia in spinal cord. **e-g** Diagrams showing Nissl + profiles per mm^2^ in (**e**) cortex and (**f**) striatum of the respective groups of mice ipsilateral and contralateral to the sciatic nerve crush injury and (**g**) ipsilateral spinal cord (mean ± SEM). **h** Number of CD11b + cells per mm^2^ of the different groups of mice in white (wm) and grey matter (gm) are shown (mean ± SEM). *n* = number of mice are shown below the diagrams. Statistical analysis was performed by using the parametric one-way ANOVA with posthoc Tukey test

Next, we examined the somatosensory cortex, striatum and the spinal cord (L2-4) for structural alterations. Nissl staining in cerebral cortex (Fig. 3a,e) striatum (Fig. 3b,f) and ipsilateral spinal cord (Fig. 3c,g) excluded gross structural differences. CD11b + microglia within the white and grey matter of the spinal cord did not show significantly different cell numbers in naïve and nerve crushed wt and *Tor1a*+/- mice (Fig. 3d,h).

### Abnormal central dopaminergic neurotransmission after sciatic nerve injury correlates with dystonia-like phenotype in *Tor1a*+/- mice

We next focused on the central dopaminergic system, since torsinA has been associated with dopaminergic neurotransmission and because striatal dopaminergic imbalance could be a cause of dystonia.

**Fig. 4:**
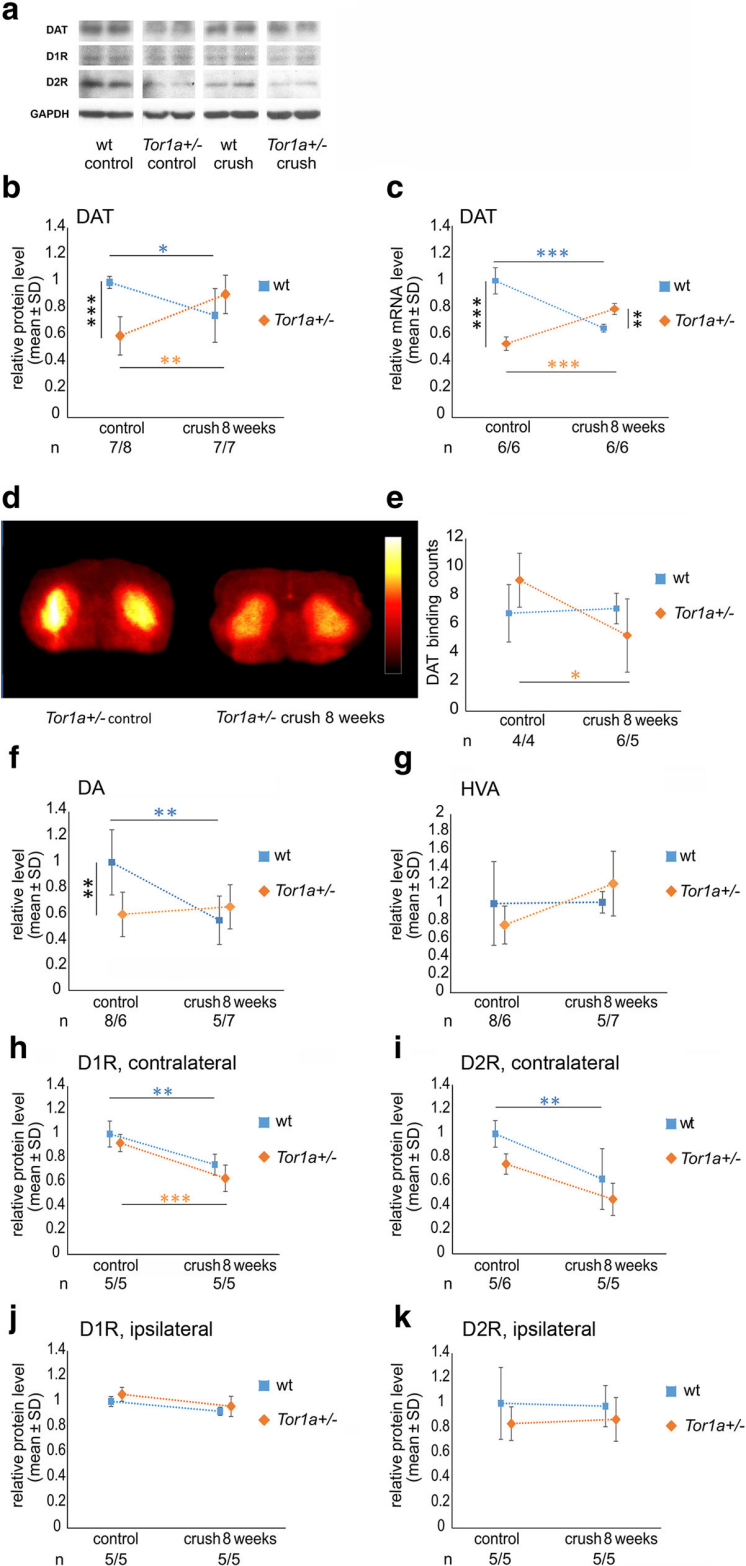
Dopamine metabolism is disturbed in Tor1a+/- mice. **a** Representative Western blot of striatal DAT, DA D1 and DA D2 receptors in naïve wt, naïve *Tor1a*+/, crush injured wt and crush injured *Tor1a*+/- mice. GAPDH is used as loading control. **b** Relative DAT, (**h**) DA D1 receptor and (**i**) DA D2 receptor protein levels in striatum contralateral to crush injury are shown (mean ± SD) comparing wt (blue) and *Tor1a*+/- (orange) mice before and 8 weeks after crush injury. **c** Diagram demonstrates relative DAT mRNA expression by real-time PCR in midbrain contralateral to crush injury in wt and *Tor1a*+/- mice before and 8 weeks after sciatic nerve crush (mean ± SD). **d** Representative images of in-vivo DAT autoradiography with FP-CIT in *Tor1a*+/- control (left) and crush injured mutant (right). **e** Diagram shows mean striatal DAT binding counts in wt and *Tor1a*+/- mice before and 8 weeks after sciatic nerve crush (mean ± SD). **f, g** Relative striatal DA and HVA levels measured by HPLC are shown in wt and *Tor1a*+/- mice before and 8 weeks after crush injury contralateral to nerve crush (mean ± SD). **j, k** Ipsilateral DA D1 and DA D2 receptor relative protein levels are demonstrated in wt and *Tor1a*+/- mice before and 8 weeks after crush injury (mean ± SD). *n* = number of mice are depicted below the diagrams (wt/*Tor1a*+/-). Statistical analysis was performed by using the parametric one-way ANOVA with posthoc Tukey test. *p < 0.05, **p < 0.01, ***p < 0.001

Untreated *Tor1a*+/- mice showed an about 40 % reduction of striatal presynaptic DA transporter (DAT) as compared to wt mice at the protein level (*p* < 0.001). Eight weeks after nerve injury, a significant decrease of DAT from baseline was observed in the striatum of wt animals contralateral to the nerve crush lesion while a significant increase of DAT protein was found in *Tor1a*+/- mice (Fig. 4a,b). Comparable changes in DAT mRNA levels were noted within the contralateral midbrain indicating that alteration in DAT expression occurs at the transcriptional level (Fig. 4c). Striatal DAT autoradiography as a measure for presynaptic dopaminergic function showed a trend but no significant elevation of DAT binding in untreated *Tor1a*+/- versus wt mice. DAT binding decreased significantly in mutant mice eight weeks after sciatic nerve crush, while no change occurred in wt (Fig. 4d,e).

Measurements of striatal DA and its metabolite homovanilic acid (HVA) by HPLC of the contralateral striatum showed an about 40 % reduction of DA level in naïve mutant mice as compared to wt mice (*p* < 0.01). Eight weeks after crush injury DA level were diminished by about 50 % in wt mice (*p* < 0.01) and appeared mildly increased in *Tor1a*+/- but this was not significant (Fig. 4f). HVA levels also only showed a trend towards a slight increase from baseline in *Tor1a*+/- mice eight weeks after crush injury which was again not significant (Fig. 4g).

We did not find any differences in DA D1 and D2 receptor protein expression in wt and mutant mice at baseline. Only after sciatic nerve crush a 25 % to 35 % (*p* < 0.01) decline of D1 and D2 receptor proteins was found in both wt and *Tor1a*+/- mice on the contralateral striatum (Fig. 4h ,i), but not in the ipsilateral striatum (Fig. 4j,k)

### Pharmacological manipulation of the dopaminergic system influences the dystonia-like phenotype in *Tor1a*+/- but not wt mice

**Fig. 5:**
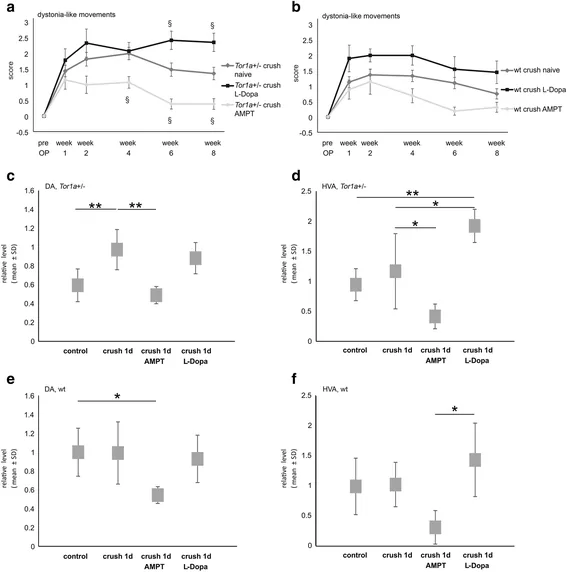
Pharmacologic modulation of the central dopaminergic system influences dystonia-like phenotype in *Tor1a+/-* but not wt mice. **a** Diagram demonstrates abnormal movements measured by the DLMS of *Tor1a* + -/ mice before and after sciatic nerve crush during an observation time of 8 weeks. 3 groups of mice are investigated: Naïve mutant mice (dark grey line; *n* = 33), *Tor1a*+/- mice treated with AMPT (light grey line; *n* = 13) and L-Dopa/benserazide (black line; *n* = 14) (mean ± SEM). **b** Focal DLM score of 3 groups of wt mice before and after sciatic nerve crush is shown (naïve mice - dark grey line, *n* = 30; AMPT treated mice – light grey line, *n* = 9; L-Dopa/benserazide treated mice – black line, *n* = 10) (mean ± SEM). § demonstrates a significant difference after Bonferroni-Holm correction of the p-values (non-parametric two-tailed Mann-Whitney test) for the whole time span of 8 weeks comparing either (**a**) naïve nerve injured *Tor1a+/-* or (**b**) wt mice with AMPT or L-Dopa/benserazide treated mice of the same genotype for all time points. **c, d** Diagrams show (**c**) relative DA and (**d**) HVA level contralateral to the crush injury side of naïve (*n* = 6) and *Tor1a*+/- mice 1 day after nerve crush (*n* = 5) as well as crush injured mutant mice that received either AMPT (4 h after injection of the last of 3 AMPT administrations, *n* = 5) or L-Dopa/benserazide treatment (90 min after injection of L-Dopa, *n* = 5) (mean ± SD). **e, f** Diagrams demonstrate (**e**) relative DA and (**f**) HVA level contralateral to the crush injury in wt mice that were treated with either AMPT (*n* = 5) or L-Dopa/benserazide treatment (*n* = 5) (mean ± SD). Statistical analysis was performed by using the parametric one-way ANOVA with posthoc Tukey test. *p < 0.05, **p < 0.01

We questioned whether the observed striatal DA dysregulation was a cause or consequence to the development of post-crush dystonia-like movements in *Tor1a+/-* mice. To answer this question we performed two additional experiments: First we induced central DA depletion using alpha-methyl-p-tyrosine (AMPT) and secondly we challenged mice with the DA precursor L-Dopa/benserazide to increase striatal DA levels. To evaluate the treatment response behavioral analyses were done and striatal DA levels were measured. Chronic treatment of *Tor1a*+/- mice with AMPT led to a significant reduction of the DLMS score starting 4 weeks after crush injury in comparison to untreated mutant mice. In contrast, repeated L-Dopa/benserazide injections induced a significant higher DLMS score in mutant mice from week six on (Fig. 5a). In wt mice however, L-Dopa or AMPT therapy did not lead to any significant changes in DLMS scores (Fig. 5b). Neurochemically, AMPT treatment resulted in a significant reduction of the elevation of DA and HVA observed in prior experiments one day after nerve crush in *Tor1a*+/- mice. L-Dopa/benserazide injection analyzed 90 min after administration even led to a further increase of HVA as compared to the one day post nerve crush situation (Fig. 5c,d). In contrast, we did not observe a significant elevation of the DA or HVA level in wt mice one day after nerve injury as compared to control mice. 90 min after L-Dopa administration, DA level in wt mice was again comparable to baseline control (Fig. 5e,f).

## Discussion

We describe an abnormal motor behavior in *Tor1a*+/- mice after peripheral nerve injury, that fits very well to the phenotypical description of dystonia based on the latest consensus definition [2]. Within the first 2 weeks after the peripheral nerve injury both wt and *Tor1a+/-* mice developed abnormal posturing and distorted movements resembling pseudodystonia described in humans after limb deafferentation [2]. At later timepoints, during sensorimotor recovery, however, the severity of dystonia-like movements was more pronounced in mutant mice and only in *Tor1a+/-* mice sensitive to pharmacological modulation of central dopaminergic neurotransmission. Moreover, in *Tor1a+/-* mice the appearance of dystonia-like movements was associated with functional impairment of the gait pattern that was still present after 8 weeks indicating a predisposition to consolidate this movement disorder. In contrast, abnormal movements in wt mice were significantly less severe and were accompanied by only transient and mild gait impairment likely due to peripheral denervation alone.

Several lines of evidence suggest that an altered dopaminergic neurotransmission in *Tor1a+/-* mice may represent the endogenous predisposition for the observed intensified and prolonged dystonia-like movements in response to nerve injury. This assumption is based on two observations: (1) *Tor1a+/-* mice were characterized by a hypodopaminergic state at baseline as compared to wt mice and (2) a paradoxical increase of dopaminergic neurotransmission was shown in response to the nerve injury as reflected by an upregulation of presynaptic DAT, an increased DA metabolism and downregulation of D1 and D2 receptors. These alterations were opposed to the striatal dopaminergic downregulation of wt mice during sensorimotor recovery from the sciatic nerve lesion. It has been shown that electrical (sensory) stimulation of the rat forepaw inhibits striatal DA release conceivably by activation of striatal GABA-ergic striatal interneurons by glutamate [11]. We assume that in wt mice at least partially comparable mechanisms led to chronic downregulation of striatal DA and DAT after nerve crush with a constant sensory stimulus due to the nerve injury during the recovery phase. We hypothesize that *Tor1a+/-* mice on the other hand present a deficit in central inhibition that drives uncontrolled central DA efflux after peripheral nerve injury. Indeed, impaired central GABA-ergic control leading to disinhibition of the sensorimotor system has been suggested as a pathogenetic mechanism of dystonia based on data from a DYT1 mouse model, the *dt* ^*sz*^ mutant hamster but also from DYT1 carriers and sporadic dystonia patients [18, 22, 47]. In the latter study a reduction in GABA receptor affinity in C11-flumazenil (a selective GABA(A) receptor ligand) positron emission tomography (PET) was observed [18].

In proof of principle experiments, we used established pharmacological treatments to suggest a causal link between the observed imbalance in the basal ganglia-thalamo-cortical network with focal dystonia-like movements in the previously injured limb. By treating *Tor1a+/-* mice with either L-Dopa or the DA synthesis blocker AMPT we could drive striatal DA concentrations into opposite directions and either aggravate or reduce dystonia-like movements in the mutant mice as compared to those observed with no treatment. In contrast, abnormal movements in wt mice did not respond significantly to dopaminergic modulation. This supports the notion that the motor symptoms in wt mice reflect peripheral pseudodystonia as result of peripheral deafferentation rather than central dystonia as the observed phenotype in mutant mice. The analyses of the dopaminergic metabolism after L-Dopa administration suggested a compensatory mechanism to preserve dopamine homeostasis and prevent a hyperdopaminergic striatal environment in wt mice as opposed to *Tor1a*+/- mice. Dopaminergic nigrostriatal input is crucial for motor function and regulates the activity in the direct and indirect basal ganglia pathway via DA D1 and D2 receptors. Alterations of neurochemical metabolism have been detected in different mouse models for DYT1 dystonia demonstrating substantial discordance concerning dopaminergic changes. Complementary to the findings in *Tor1a+/-* mice, DYT1 knock-down mice showed a slight but not significant decrease in striatal DA while in a transgenic DYT1 mouse model containing human wt torsinA striatal DA was significantly reduced [15, 21]. However, in contrast to our findings in *Tor1a+/-* mice a DYT1 transgenic model overexpressing human ∆E-torsinA developed increased striatal DA levels by 18 % in asymptomatic and a decrease by 39 % in symptomatic animals showing abnormal movements [50]. DYT1 knock-in mice and hΔGAG transgenic mice on the other hand did not exhibit changes in striatal DA and DOPAC level [14, 21].

In principle, the role of striatal dopaminergic neurotransmission as an important modifier for the manifestation of dystonia in a multistep pathophysiological pathway is widely accepted already because inherited defects of DA synthesis or acquired alterations of DA neurotransmission, such as in Parkinson’s disease or with pharmacotherapy altering dopaminergic neurotransmission [10, 42] are well known causes of dystonia. Moreover, mutations in genes involved in the biosynthesis of DA encoding GTP cyclohydroxylase (DYT5), sepiapterin reductase and tyrosine hydroxylase cause dopa-responsive dystonia [27, 32]. Additionally, *GNAL* (DYT25) that encodes the stimulatory α subunit of a G-protein (Gαolf), has also been linked to DA signaling because it was shown to be necessary for D1 receptor coupling [13]. A novel finding in our experiments, however, is the abnormal dynamic response of striatal DA neurotransmission in *Tor1a*+/- mice during sensorimotor recovery from a peripheral nerve injury, which may offer an attractive link between inherited predisposition and environmental triggers of DYT1 dystonia. Dopaminergic nigrostriatal input regulates the activity in the direct and indirect basal ganglia pathway via D1 and D2 receptors. Indeed, the nigrostriatal dopaminergic fibers terminate on the shafts of the dendritic spines of the medium spiny neurons (MSN) and the cortical afferents terminate on the heads of spines, enabling DA modulation of the corticostriatal input. Alterations of DA dependent synaptic plasticity in medium spiny neurons have been implicated in the basal ganglia network dysfunction associated with several psychiatric or neurological diseases [7], in particular L-Dopa induced dyskinesia in Parkinson’s disease [10]. A common disease mechanism could be the inability of the striatum to filter neuronal signals once the activities of direct and indirect basal ganglia pathways become unbalanced due to abnormal DA-mediated MSN function [7].

The molecular mechanism of an abnormal striatal DA homeostasis in *Tor1a*+/- mice could not be addressed by our methods, but an increased dopaminergic neurotransmission in response to the nerve crush against the background of a baseline hypodopaminergic state resembles the mechanism of dyskinesia development in Parkinson’s disease. Dyskinesia are thought to result from sensitization of postsynaptic DA receptors through large swings in striatal extracellular DA concentration with fluctuating L-Dopa plasma levels [10]. Presynaptic mechanisms, in particular DAT reuptake, play an important role in maintaining an extracellular DA homeostasis [1] and the reduced availability of DAT with presynaptic degeneration is one major cause of pulsatile dopaminergic neurotransmission in Parkinson’s disease [10]. Indeed, although DAT protein levels were increased in *Tor1a*+/- mice after crush injury, our autoradiography data suggest a malfunction of DAT with impaired capability to utilize DA since DAT-ligand binding by FP-CIT was significantly lower after crush injury than before. Another possible explanation why a 50 % reduced torsinA level in *Tor1a*+/- mice might affect DAT function could be a reduced availability of DAT at presynaptic membranes. TorsinA has been shown to regulate cellular trafficking of DAT to the plasma membrane thus affecting DA uptake [54]. A defective DA reuptake however, is not uniformly described. One DYT1 mouse model did not present any changes in striatal DAT or D1/D2 receptor binding- Yet, these animals still presented with an attenuated motor response to amphetamine administration suggesting a DA release problem [5]. Presynaptic release deficits of neurotransmitter have also been observed in brain slices of a DYT1 mouse model [58]. Other studies emphasized a disturbed D1 and D2 receptor function in DYT1 models [47, 48, 59] linking the D2-receptors to disinhibition of striatal GABAergic synaptic activity [47] and imbalanced dopaminergic to cholinergic signaling [48].

Collectively, a wealth of preclinical data links DYT1 dystonia to impaired striatal DA transmission and this is in keeping with our findings in mutant mice. Still, the exact synaptic mechanisms need to be addressed in future studies. Clinical data also suggest dopaminergic disturbances in dystonia. Radioligand neuroimaging of the dopaminergic system in patients with isolated dystonia of mixed etiology revealed reduced D2 receptor binding and mildly reduced DA metabolism in [^18^F]Dopa PET [35, 41, 43]. *DYT1* mutation carriers also presented reduced striatal D2 receptor binding compared to normal controls [3]. The few available post mortem analyses of DYT1 brains though provided only subtle evidence of impaired DA neurotransmission, such as a mild reduction of striatal DA level [17] or a higher DA turnover in the striatum [4]. However, in keeping with our observation of an increase in DA neurotransmission in nerve injured, dystonic *Tor1a*+/- mice approaching wt baseline level, one would expect none or only mild DA metabolic changes comparing dystonia manifesting DYT1 patients with healthy subjects. DA neurotransmission changes in dystonia are likely just one step within a complex cascade of secondary maladaptive plasticity of the central network in *Tor1a+/-* mice. Indeed, deficits of striatal synaptic plasticity with loss of inhibition have been found in DYT1 mouse and rat models [20, 33] and these were connected to partial D2 receptor dysfunction [33, 34]. Furthermore maladaptive motor cortical plasticity has been correlated to dystonia in patients with task-specific focal dystonia like writer’s cramp and musician’s dystonia [23, 44]. In addition FDG-PET studies on DYT1 patients revealed metabolic network abnormalities in basal ganglia, cerebellum and motor areas [9, 16] and similar alterations in cerebral glucose metabolism were also found in *Tor1a*+/- mice [56]. Triggering dystonia in a DYT1 knock-in model using mitochondrial complex-II inhibition as a metabolic stressor has not been successful [6]. In contrast, the nerve injury in our present experiments leads to compensatory neuronal plasticity [36, 40]. Thus, we aimed to disturb central sensorimotor integration and to challenge the adaptive capabilities of the central motor network.

## Conclusions

In summary, we were able to link the genetic defect of reduced torsinA expression in a DYT1 related mouse model to a maladaptive response of the striatal dopaminergic system after a peripheral nerve lesion and to the manifestation of dystonia-like movements. Our findings raise the interesting possibility, that preventive antidopaminergic treatment could reduce the risk of manifesting dystonia in patients carrying the DYT1 mutation.

## Additional files

Additional file 1: Figure S1. Picture of PCR genotyping results and table demonstrating numbers and gender of animals for each experiment. (a) Results from genomic PCR analysis of 9 representative animals. On the left side 100 bp DNA ladders are visible. In the upper panel all analyzed mice show a wt band at 308 bp. In the lower panel an additional knockout band at 1600 bp is visible in 4 animals (*Tor1a+/-*). (b) Table shows the number and gender of mice used for the experiments. (TIF 14399 kb)

Additional file 2: Suppl. video dystonia score 0. Video of a mouse during tail suspension test demonstrating no dystonia-like movements (DLMS 0). (MP4 2022 kb)

Additional file 3: Suppl. video dystonia score 1. Video of a mouse during tail suspension test showing slight dystonia-like movements (DLMS 1). (MP4 1004 kb)

Additional file 4: Suppl. video dystonia score 2. Video of a mouse during tail suspension test demonstrating a DLMS of 2. (MP4 1744 kb)

Additional file 5: Suppl. video dystonia score 3. Video of a mouse during tail suspension test demonstrating a DLMS of 3. (MP4 929 kb)

Additional file 6: Suppl. video dystonia score 4. Video of a mouse during tail suspension test demonstrating a DLMS of 4. (MP4 927 kb)
