# Supplementary figures and images for: Tor1a+/- mice develop dystonia-like movements via a striatal dopaminergic dysregulation triggered by peripheral nerve injury
Source: Acta Neuropathol Commun. 2016 Oct 3;4:108. doi: 10.1186/s40478-016-0375-7 (PMC5048687; doi:10.1186/s40478-016-0375-7)

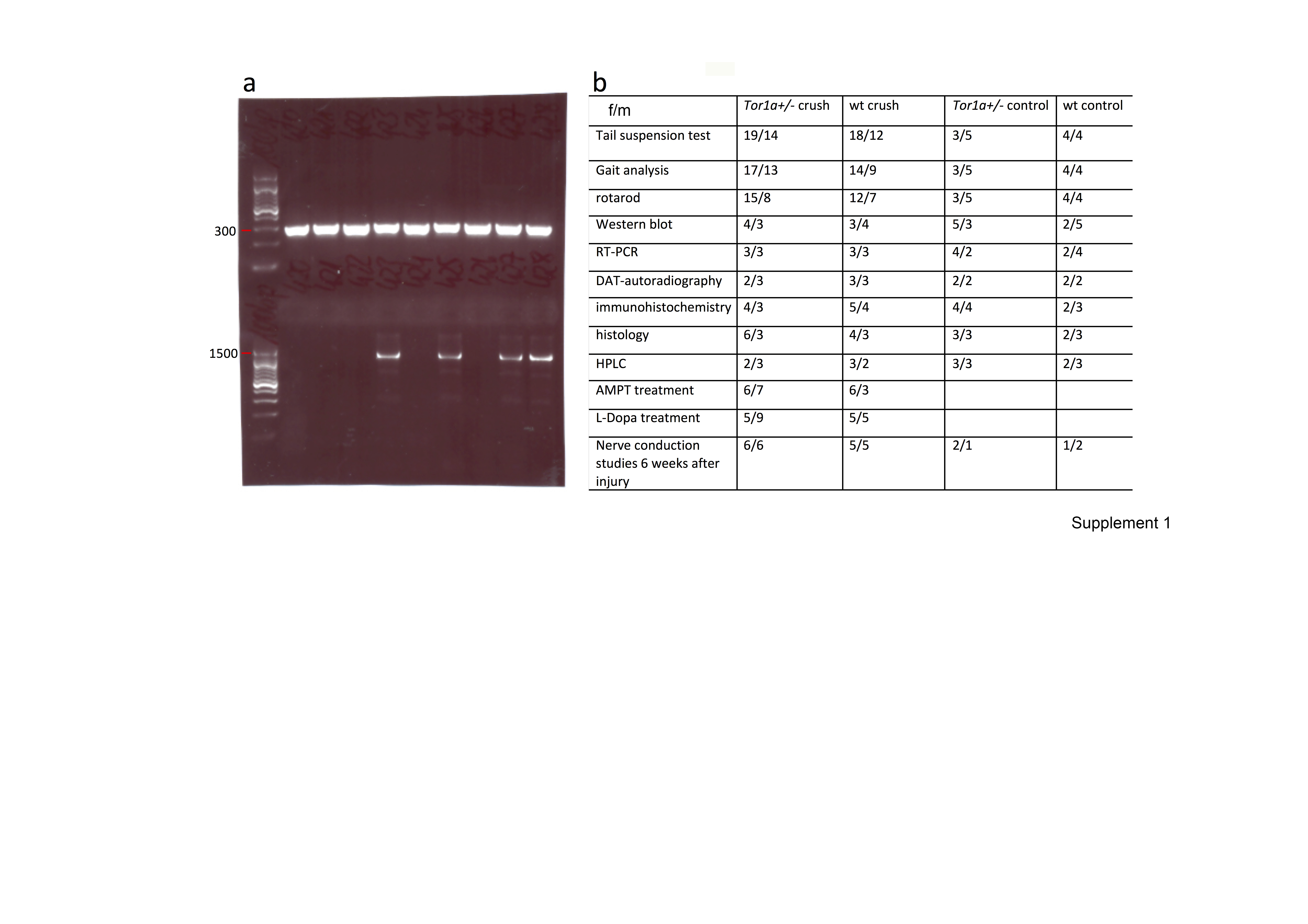

Supplement: Additional file 1: Figure S1. — Picture of PCR genotyping results and table demonstrating numbers and gender of animals for each experiment. (a) Results from genomic PCR analysis of 9 representative animals. On the left side 100 bp DNA ladders are visible. In the upper panel all analyzed mice show a wt band at 308 bp. In the lower panel an additional knockout band at 1600 bp is visible in 4 animals (Tor1a+/-). (b) Table shows the number and gender of mice used for the experiments. (TIF 14399 kb) [file 40478_2016_375_MOESM1_ESM.tif]
